# Adiponectin Protein Exists in Aortic Endothelial Cells

**DOI:** 10.1371/journal.pone.0071271

**Published:** 2013-08-13

**Authors:** Noriyuki Komura, Norikazu Maeda, Takuya Mori, Shinji Kihara, Hideaki Nakatsuji, Ayumu Hirata, Yoshihiro Tochino, Tohru Funahashi, Iichiro Shimomura

**Affiliations:** 1 Department of Metabolic Medicine, Graduate School of Medicine, Osaka University, Suita, Osaka, Japan; 2 Department of Biomedical Informatics, Graduate School of Medicine, Osaka University, Suita, Osaka, Japan; 3 Department of Metabolism and Atherosclerosis, Graduate School of Medicine, Osaka University, Suita, Osaka, Japan; University of Sao Paulo, Brazil

## Abstract

**Aims:**

Inflammation is closely associated with the development of atherosclerosis and metabolic syndrome. Adiponectin, an adipose-derived secretory protein, possesses an anti-atherosclerotic property. The present study was undertaken to elucidate the presence and significance of adiponectin in vasculature.

**Methods and Results:**

Immunofluorescence staining was performed in aorta of wild-type (WT) mice and demonstrated that adiponectin was co-stained with CD31. Thoracic aorta was cut through and then aortic intima was carefully shaved from aorta. Western blotting showed the existence of adiponectin protein in aortic intima, while there was no adiponectin mRNA expression. Adiponectin knockout (Adipo-KO) and WT mice were administered with a low-dose and short-term lipopolysaccharide (LPS) (1 mg/kg of LPS for 4 hours). The endothelium vascular adhesion molecule-1 (VCAM-1) and intercellular adhesion molecule-1 (ICAM-1) were highly increased in Adipo-KO mice compared to WT mice after LPS administration.

**Conclusions:**

Adiponectin protein exists in aortic endothelium under steady state and may protect vasculature from the initiation of atherosclerosis.

## Introduction

Obesity is the most common nutritional disorder in the industrial countries and is the common basis of atherosclerotic cardiovascular diseases [1]. Our group previously identified adiponectin in human adipose tissues [2] and adiponectin is characterized by its paradoxical decrease in obesity in spite of adipose-specific secretory protein [3]. Adiponectin plays a central role in the development of metabolic syndrome and atherosclerosis [4]. In epidemiologic studies, we and others have suggested that low level of plasma adiponectin is closely associated with cardiovascular diseases and metabolic syndrome [5]–[8].

Chronic low-grade inflammation is closely associated with the development of metabolic syndrome and atherosclerosis [9]. Endotoxin (lipopolysaccharide, LPS) is one of the potent virulence factors of Gram-negative bacterial species and plays a major role in both acute and chronic infections. Several studies reported that the exposure to LPS induces systemic inflammation, leading to the development of obesity-related disorders such as diabetes and atherosclerosis [10]–[15]. High level of serum LPS may be one of risk factors for the development of atherosclerosis. LPS also accelerates the initial step of atherosclerosis through the increase of endothelial adhesion molecules. The *in vitro* and *in vivo* experiments have shown anti-atherogenic properties of adiponectin [6], [16]–[18]. Among *in vitro* experiments, adiponectin attractively suppressed the attachment of monocytes to endothelial cells, suggesting that adiponectin inhibits the initial step of atherosclerosis [6], [18]. However, little is known about the *in vivo* effect of adiponectin on LPS-induced increase of endothelial adhesion molecules in vasculature.

Adiponectin protein was detected in heart tissue, kidney, and vasculature when these organs were injured or overloaded [17], [19]–[22], while the molecular mechanism for accumulation of adiponectin in the injured tissues has remained uncertain. However, there was no evidence for the existence of adiponectin protein on vasculature under steady state, because perivascular fat tissues express adiponectin and are contained in whole aorta sample. In present study, we developed the procedure to remove mouse aortic intima from whole aorta, measured adiponectin protein in aortic intima, and examined the effect of adiponectin-deficiency on LPS-induced increase of endothelial adhesion molecules.

## Materials and Methods

### Animals

Adiponectin knockout (Adipo-KO) mice were generated and backcrossed as described previously [23]. Wild-type (WT) and Adipo-KO mice were intraperitoneally injected with 1 mg/kg of LPS (L4391, Sigma-Aldrich, St. Louis, MO) at 10 weeks of age. At 4 hours after saline or LPS administration, mice were anesthetized by intraperitoneal injection of medetomidine (0.3 mg/kg body wt), midazolam (4 mg/kg body wt), and butorphanol (5 mg/kg body wt) before euthanization. To monitor the adequacy of anesthesia, we carefully tested for no spontaneous movements of mice tail by mild stimulation. The experimental protocol was approved by the Ethics Review Committee for Animal Experimentation of Osaka University School of Medicine. This study also conforms to the Guide for the Care and Use of Laboratory Animals published by the US National Institutes of Health (NIH Publication No. 85-23, revised 1996).

### Immunofluorescence Staining

Double-immunofluorescence method was performed to identify the localization of adiponectin and CD31 in sections of aorta. Aortas were embedded and frozen in Tissue-Tek O.C.T. Compound (Sakura Finetek, Torrance, CA), and subsequently cut at 6-µm sections and mounted on glass slides by the standard procedures. Briefly, sections were fixed in ice-cold acetone for 20 min and washed for 5 min in PBS (pH 7.4). Sections were stained with antibodies against rabbit anti-adiponectin (Otsuka Pharmaceutical, Tokushima, Japan) and rat anti-CD31 (BD Biosciences, San Jose, CA) or control rabbit and rat IgG. The secondary antibody was goat anti-rabbit IgG conjugated Alexa 488 (Life Technologies, Gaithersburg, MD) and goat anti-rat IgG conjugated Alexa 594 (Life Technologies, Gaithersburg, MD). Cell nuclei were counterstained with DAPI, and slides were imaged on Olympus FV1000D confocal laser scanning microscope system.

### Western Blotting

For the collection of aortic intima, mice were transcardially perfused with cold saline to eliminate the contamination of circulating adiponectin. After removing perivascular fat, thoracic aorta was cut through and then intima was carefully shaved from the aorta (Figure S1). The excised aortic intima was washed twice with PBS and then was sonicated in lysis buffer (50 mmol/L HEPES (pH7.8), 1% Triton X100, 10% Glycerol, 10 mmol/L EDTA (pH8.0), 10 mmol/L Sodium Diphosphate Decahydrate, 1% NP-40, 100 mmol/L NaF, 5 mmol/L Na_3_VO_4_, 10 µg/mL aprotinin, 5 µg/mL leupeptin, 1.5 mg/mL benzamidine, and 100 mmol/L phenylmethylsulfonyl fluoride (PMSF)) followed by centrifugation.

Equal amount of proteins were separated by 5–20% gradient SDS-PAGE gel and transferred onto nitrocellulose membranes (GE Healthcare, Piscataway, NJ). The following primary antibodies were used and signals were detected by ECL Western Blotting Detection System (GE Healthcare, Piscataway, NJ): anti-mouse adiponectin (Otsuka Pharmaceutical, Tokushima, Japan), anti-mouse perilipin A antibody (Sigma-Aldrich, St Louis, MO), anti-mouse CD31 antibody (BD Biosciences, San Jose, CA), anti-mouse β-actin antibody (Cell Signaling, Danvers, MA), and anti-mouse vascular adhesion molecule-1 (VCAM-1) antibody (R&D Systems, Minneapolis, MN).

For the analysis of oligomeric adiponectin complexes, samples were incubated with a non-reducing sample buffer containing 2% SDS, 10% glycerol, and 10 mmol/L Tris-HCl (pH6.8) at room temperature for 10 min, separated by a 5–20% gradient SDS-PAGE, and immunoblotted with the indicated antibodies as previously described [24].

### Quantification of mRNA Levels

Total RNA was isolated from mice tissues by using RNA STAT-60 (Tel-Test Inc., Friendswood, TX) according to the protocol supplied by the manufacturer. The quality of total RNA was determined by using ND-1000 spectrophotometer (Nano Drop Technologies, Wilmington, DE). First-strand cDNA was synthesized from 180 ng of total RNA using Thermoscript RT (Invitrogen Corp., Carlsbad, CA) and oligo(dT) primer. Real-time quantitative polymerase chain reaction amplification was conducted with the LightCycler 1.5 (Roche Diagnostics, Tokyo, Japan) using LightCycler-FastStart DNA Master SYBR Green I (Roche Diagnostics, Tokyo, Japan) according to the protocol recommended by the manufacturer. Primer sets were as follows: mouse adiponectin, 5′-GATGGCAGAGATGGCACTCC-3′ and 5′-CTTGCCAGTGCTGCCGTCAT-3′; mouse vascular adhesion molecule-1 (VCAM-1), 5′-CTTCATCCCCACCATTGAAG-3′ and 5′-TGAGCAGGTCAGGTTCAGAG-3′; mouse intercellular adhesion molecule-1 (ICAM-1), 5′-AACCGCCAGAGAAAGATCAG-3′ and 5′-TGTGACAGCCAGAGGAAGTG-3′; mouse β-actin, 5′-CCTGAGGCTCTTTTCCAGCC-3′ and 5′-TAGAGGTCTTTACGGATGTCAACGT-3′. The final result for each sample was normalized to the respective β-actin value.

### Laboratory Methods

Protein concentrations in tissues and cells were determined by the BCA assay (Thermo Fisher Scientific, Rockford, IL).

### Statistical Analysis

Data are presented as mean±SEM. Differences between groups were evaluated by the unpaired Student’s *t*-test or analysis of variance followed by Fisher PLSD test. A probability value less than 0.05 denoted the presence of a statistically significant difference. All calculations were performed by using JMP version 9.

## Results

### Immunohistochemical Detection of Adiponectin in Aorta

**Figure 1:**
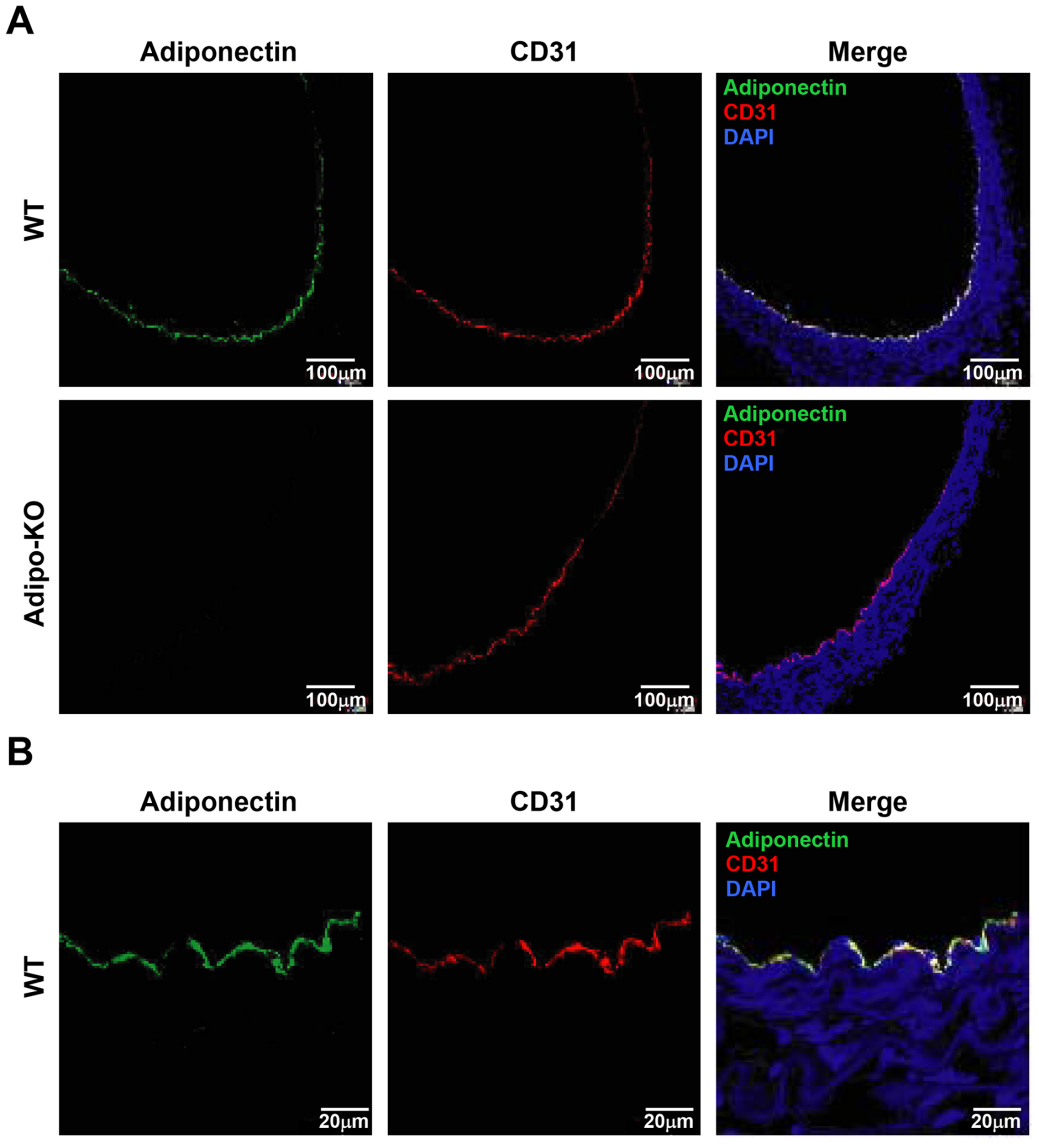
Localization of adiponectin in aorta. A, Dual-immunofluorescence was performed in wild-type (WT) and adiponectin knockout (Adipo-KO) mice as described in Materials and Methods. B, High magnification images of aortic intima using confocal laser microscope were obtained from WT mice. Green, adiponectin; blue, DAPI; red, CD31.

Firstly, immunohistochemical analysis was performed to investigate the localization of adiponectin protein in mice aorta. Dual-immunofluorescence staining was performed on adiponectin (green) or CD31 (red), an endothelial cell marker. Adiponectin was detected in aortic intima of wild-type (WT) mice and was co-stained with CD31 (Figure 1A upper panels), while adiponectin staining was absent in aortic intima of Adipo-KO mice as negative control (Figure 1A lower panels). Secondly, we examined the aortic endothelial localization of adiponectin in WT mice with confocal microscopy. Adiponectin staining was mainly confined to CD31, suggesting that adiponectin protein was existed at endothelial cells (Figure 1B).

### Adiponectin Protein in Aortic Intima

**Figure 2:**
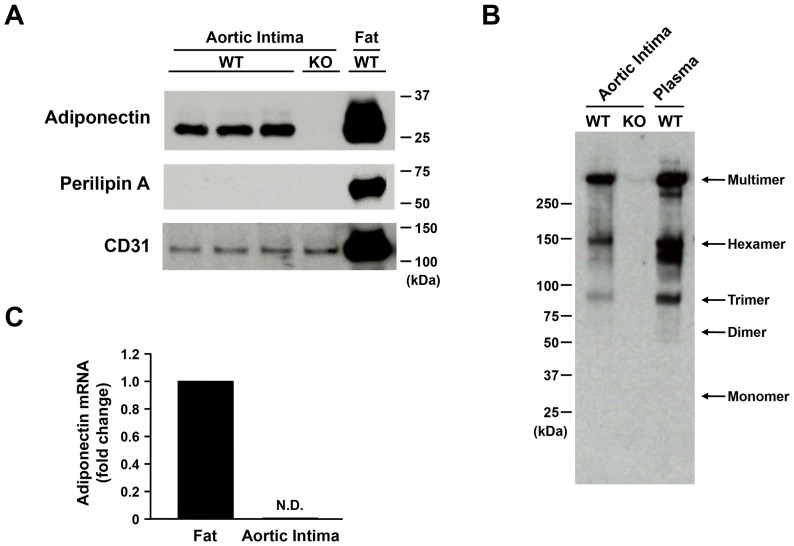
Existence of adiponectin protein in aortic intima. Mice were perfused with cold saline to eliminate the contamination of circulating adiponectin. After removing perivascular fat, thoracic aorta was cut through and then intima was carefully shaved from the aorta. A, Western blotting with antibodies against adiponectin, perilipin A, and CD31 in aortic intima. B, Multimeric complexes of adiponectin protein in aortic intima. C, The mRNA level of adiponectin in fat tissue and aortic intima. WT, wild-type mice; KO, adiponectin knockout mice; N.D., not detected.

To quantify the amount of adiponectin protein in aortic intima, we separated the intima from aorta as described in Materials and Methods section (Figure S1) and performed western blotting (Figure 2A). Adiponectin protein existed in the aortic intima of WT mice, but not in Adipo-KO mice (Figure 2A). Perilipin A, a specific protein of adipocytes, was not detected in aortic intima of both WT and Adipo-KO mice, indicating that adipose tissues were not contaminated in the intima section. CD31 protein levels were equally detected both in aortic intima of WT and Adipo-KO mice (Figure 2A). Non-heating and non-reducing SDS-PAGE analysis of aortic intima resolved into three distinct bands, that represent the three oligomeric forms of adiponectin (Figure 2B). The upper band, with an apparent molecular weight of >250 kDa, is equivalent to high molecular weight (HMW)-adiponectin. The middle band (∼180 kDa) and the lower band (∼90 kDa) are equivalent to middle molecular weight (MMW)- and low molecular weight (LMW)-adiponectin, respectively. Isoforms of adiponectin on the aortic intima were equivalent to that in plasma. Adiponectin mRNA was not detected in aortic intima (Figure 2C), indicating that adiponectin is not produced by aortic endothelial cells.

### LPS-induced Expressions of Endothelial Adhesion Molecules under Adiponectin-deficiency

**Figure 3:**
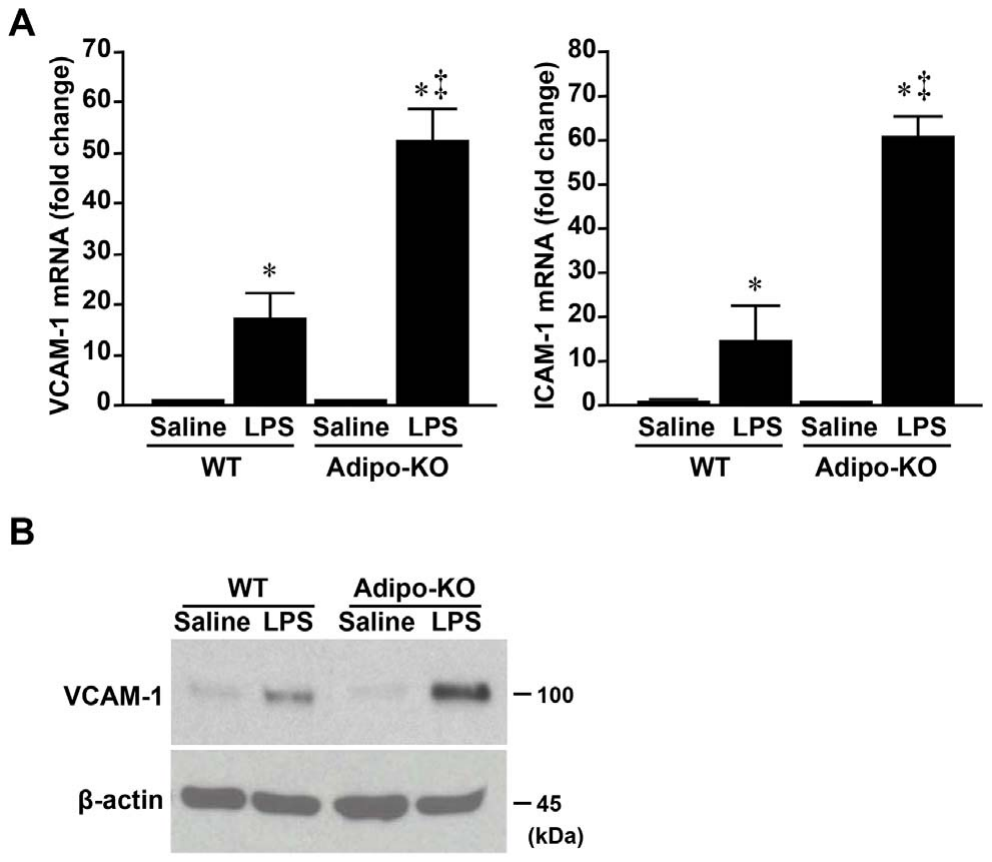
Effect of LPS on endothelial adhesion molecules in aortic intima. Aortic intima of wild-type (WT) or adiponectin knockout (Adipo-KO) mice were removed at 4 hours after LPS administration and were subjected to RT-PCR (A) and western blotting (B). Values are mean ± SEM; n = 6 for each group. * *P*<0.05, compared with the values of WT mice with saline treatment; ‡ *P*<0.05, compared with the values of WT mice with LPS administration group.

We next investigated LPS-induced vascular inflammatory response by using Adipo-KO mice. The mRNA levels of VCAM-1 and ICAM-1 were examined in aortic intima of WT and Adipo-KO mice at 4 hours after LPS administration (Figure 3). In WT mice, VCAM-1 and ICAM-1 mRNA levels were significantly increased in LPS administration group compared to saline group (VCAM-1, 16.9-fold increase from saline control group; ICAM-1, 14.5-fold increase from saline control group, Figure 3A, lane 1 versus 2). In Adipo-KO mice, mRNA levels of these endothelial adhesion molecules were more significantly increased when mice were administered with LPS (VCAM-1, 53.6-fold increase from saline control group; ICAM-1, 81.2-fold increase from saline control group, Figure 3A, lane 3 versus 4). Consequently, adiponectin-deficiency resulted in further increases of these mRNA levels under LPS administartion (Figure 3A, lane 2 versus 4). As shown in Figure 3B, amount of aortic intimal VCAM-1 protein was higher in Adipo-KO mice than in WT mice when mice were administered with LPS.

## Discussion

The major findings of the present study were as follows: (1) Adiponectin protein existed in endothelial cells of aortic intima at the steady state. (2) Adiponectin-deficiency caused significant increases of endothelial adhesion molecule expression under LPS administration.

Our previous studies demonstrated that adiponectin was immunohistochemically detected in pathological changes such as atherosclerotic lesion [17] and wire-injured vessels [22]. However, there was no distinct evidence for the existence of adiponectin protein on vasculature under steady state, because of the contamination of perivascular fat tissues expressing adiponectin. In present study, we could detect adiponectin at the endothelial cells of intact vascular walls with a confocal microscopy system. Furthermore, our precise procedure for the collection of aortic intima could eliminate the contamination of adiponectin from perivascular adipose tissues and blood stream as described in Materials and Methods section (Figure S1). Therefore, aortic endothelial adiponectin protein should be derived from bloodstream and attach to vascular endothelium. We also examined mRNA expression levels of adiponectin receptors as reported previously [25], [26], while neither AdipoR1, AdipoR2, nor T-cadherin mRNA levels in aortic intima was altered between saline and LPS groups (data not shown). Existence of endothelial adiponectin protein will be examined by using mice lacking AdipoR1, AdipoR2, or T-cadherin and the molecular interaction of adiponectin and endothelial cells remains to be clarified in the future.

Previous study showed that adiponectin accumulated at the endothelium of ischemic cortex [27] and pulmonary vessels under hypoxia [28], but immunohistochemical signal of adiponectin was hardly detected in such vessels under steady conditions. Zhang et al demonstrated that adiponectin protein in vasculature was reduced in obese *db/db* mice and its reduction was reversed by neutralizing antibody to tumor necrosis factor-α (TNF-α) [29], suggesting that TNF-α directly or indirectly suppressed vascular adiponectin protein. As shown in Figure 3, adiponectin-deficiency caused the significant increases of VCAM-1 and ICAM-1 in aortic intima under short-term LPS administration. Higher serum LPS activity was associated with obesity and chronic inflammation [10], [11]. Taken together, there is a possibility that, in obesity, the reduction of endothelial adiponectin may initiate atherosclerotic process partly independent of plasma adiponectin level. Further investigations should be performed to clarify whether obesity-associated increase of LPS relates to atherosclerosis through endothelial adiponectin.

Our previous study demonstrated that adiponectin treatment dose-dependently inhibited the TNF-α-induced monocyte adhesion to endothelial cells and expressions of VCAM-1 and ICAM-1 in human aortic endothelial cells (HAECs) [24], but such protective role of adiponectin for endothelium has not been clarified *in vivo* mouse model. We herein for the first time showed that short-term LPS administration caused the significant increase of VCAM-1 and ICAM-1 in aortic intima of Adipo-KO mice compared to WT mice (Figure 3). Several studies demonstrated that LPS induces the attachment of monocytes to endothelium in parallel with the elevation of endothelial adhesion molecules [15], [30]. Taken together, endothelial adiponectin may protect vasculature from the initiation of atherosclerotic inflammatory process.

## Supporting Information

Figure S1 **Collection of mouse aortic intima.** Mice were transcardially perfused with cold saline to eliminate the contamination of circulating adiponectin and then thoracic aorta was cut through (A). After unfolding the aorta, intima was carefully shaved from the aorta (B).
(TIF)
