# Supplementary figures and images for: Adiponectin Protein Exists in Aortic Endothelial Cells
Source: PLoS One. 2013 Aug 13;8(8):e71271. doi: 10.1371/journal.pone.0071271 (PMC3742782; doi:10.1371/journal.pone.0071271)

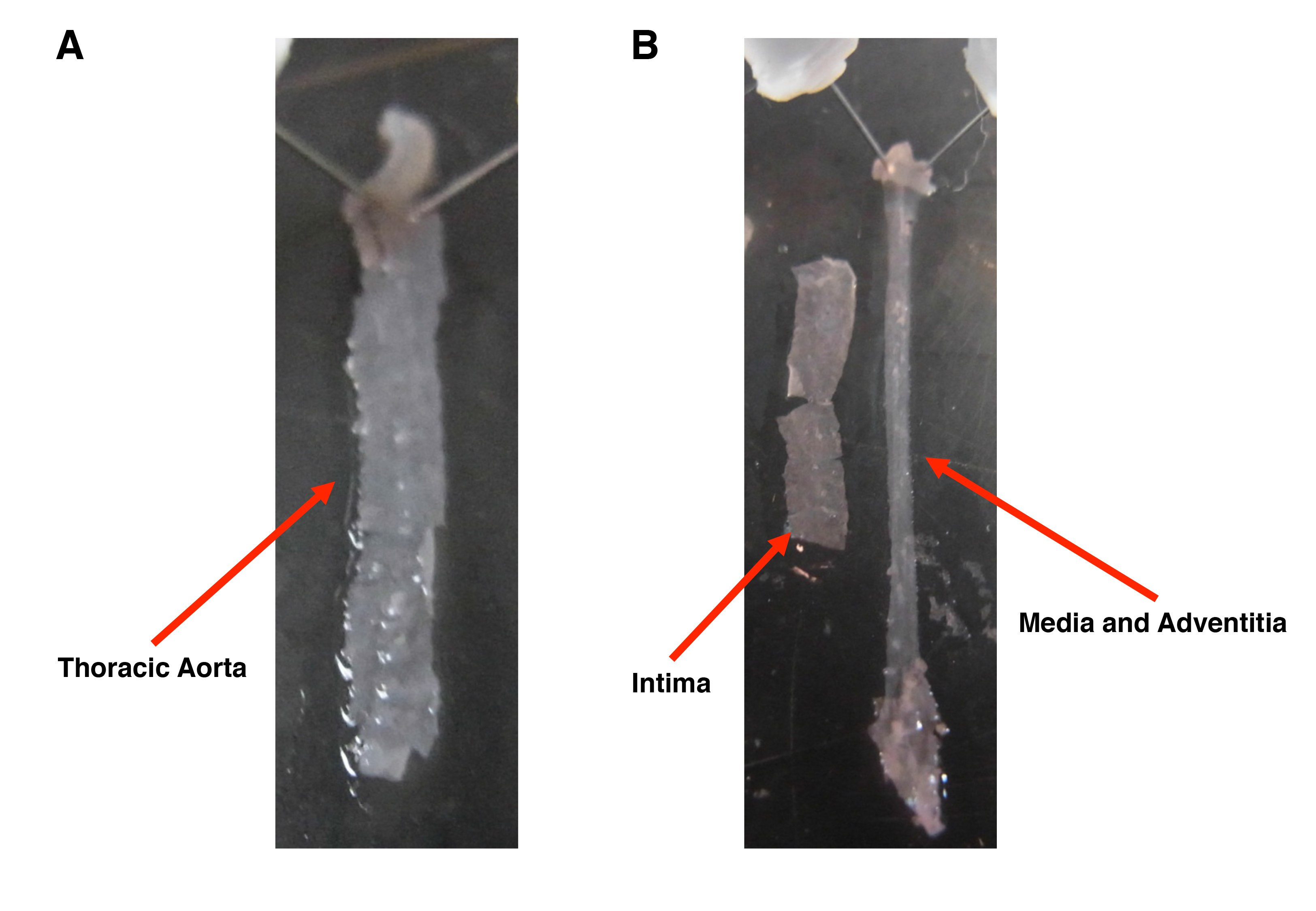

Supplement: Figure S1 — Collection of mouse aortic intima. Mice were transcardially perfused with cold saline to eliminate the contamination of circulating adiponectin and then thoracic aorta was cut through (A). After unfolding the aorta, intima was carefully shaved from the aorta (B). (TIF) [file pone.0071271.s001.tif]
